# Complete Genome Sequence of *Gordonia terrae* 3612

**DOI:** 10.1128/genomeA.01058-16

**Published:** 2016-09-29

**Authors:** Daniel A. Russell, Carlos A. Guerrero Bustamante, Rebecca A. Garlena, Graham F. Hatfull

**Affiliations:** Department of Biological Sciences, University of Pittsburgh, Pittsburgh, Pennsylvania, USA

## Abstract

Here, we report the complete genome sequence of *Gordonia terrae* 3612, also known by the strain designations ATCC 25594, NRRL B-16283, and NBRC 100016. The genome sequence reveals it to be free of prophage and clustered regularly interspaced short palindromic repeats (CRISPRs), and it is an effective host for the isolation and characterization of *Gordonia* bacteriophages.

## GENOME ANNOUNCEMENT

*Gordonia* spp. are ubiquitous slightly acid-fast nocardioform bacteria classified within the order *Actinomycetales* (1). *Gordonia* species are important components of wastewater treatment systems (2) and are noted for their biodegradation abilities (3–5) and as opportunistic pathogens (6–8). *Gordonia terrae* 3612 is a type strain maintained by ATCC (ATCC 25594), NRRL (NRRL B-16283), and NBRC (NBRC 100016). *G. terrae* 3612 has been explored as a host for bacteriophage isolation within the Science Education Alliance Phage Hunters Advancing Genomics and Evolutionary Science (SEA-PHAGES) program (9, 10), and >250 individual phages have been isolated, 80 of which have been genomically characterized (http://phagesdb.org/hosts/strains/6/). Although two whole-genome shotgun (WGS) sequencing projects have been published for *G. terrae* 3612 (accession numbers NZ_JNXA00000000 and BAFD00000000), we report here the complete and finished genome sequence for this bacterium.

Genomic DNA was obtained by lysing saturated cultures of *Gordonia terrae* 3612 in a 3110BX Mini BeadBeater for 45 s, treating with RNase, and then performing a phenol-chloroform extraction. A sequencing library was prepared using an Illumina TruSeq Nano DNA kit and was run on an Illumina MiSeq, yielding ~6.7 million 150-bp reads, representing ~175-fold coverage of the genome. These reads were assembled using Newbler 2.9 into 214 contigs, 103 of which were discarded due to very low coverage or assembly errors. A second sequencing library was prepared using an Illumina Nextera mate-pair kit, and a MiSeq run yielded ~2.9 million paired-end 300-bp reads, or an additional ~250-fold coverage of the genome. As expected, insert sizes from this run reached as high as 12 kbp, and the paired reads provided linking information to assemble the remaining contigs in the appropriate order and resolve repetitive (rRNA, transposons, etc.) regions of the genome.

The genome of *Gordonia terrae* 3612 is a single circular molecule of 5,701,501 bp, with a G+C content of 67.8%. Its closest completely sequenced relative in GenBank is *Gordonia* sp. strain KTR9, with a query coverage of 83% using standard blastn parameters. Unlike KTR9, which has three reported plasmids, we did not find any plasmids associated with 3612. The genome of 3612 was annotated using NCBI’s Prokaryotic Genome Annotation Pipeline (PGAP) and contains 4,939 predicted protein-coding genes, 46 tRNA genes, and three rRNA operons. No intact prophages were found following a PHASTER (11) search, and CRISPRFinder (12) did not reveal any likely clustered regularly interspaced short palindromic repeat (CRISPR) systems.

Notably, *G. terrae* 3612 lacks a putative prophage that is present in *G. terrae* KTR9 and is integrated at a tRNA^Ala^ gene (KTR9_RS07590). This prophage is approximately 52.7 kbp long and includes 79 predicted genes, some of which are sequence related to phages isolated on *G. terrae* 3612 (http://phagesdb.org/hosts/strains/6/). *G. terrae* 3612 contains a cognate tRNA^Ala^ gene, but the *attB* site is not occupied. Although no complete restriction modification systems were identified in *G. terrae* 3612, we note that BCM27_13045 codes for a putative modification subunit. It is plausible that *G. terrae* 3612 encodes an active restriction system, although if so, it is not an impediment to phage isolation.

### Accession number(s).

The complete *Gordonia terrae* 3612 sequence and annotation are available in GenBank with accession no. CP016594. Sequencing reads are available from the Sequencing Read Archive with accession numbers SRR3931886 and SRR3931887.
